# A novel ELISA method to determine human MrgX2 in chronic urticaria

**DOI:** 10.1186/s13601-020-00361-8

**Published:** 2020-12-09

**Authors:** Yuanyuan Ding, Tao Zhang, Rui Liu, Delu Che, Nan Wang, Langchong He

**Affiliations:** College of Pharmacy, Xi’an Jiaotong University, Yanta West Road, Xi’an, 710061 China

**Keywords:** MrgX2, ELISA, Chronic urticaria

## Abstract

**Background:**

Mas-related G-protein coupled receptor member X2 (MrgX2) directly mediates drug-induced pseudo allergic reactions. Skin mast cell MrgX2 is upregulated in severe chronic urticaria (CU). Mast cells and leukocytes are key effector cells in allergic reactions and undergo degranulation upon stimulation. It is unknown whether circulating MrgX2 expression can be detected occurs in the whole blood of CU patients and reflects pseudo-allergic reaction. There is no effective method for its detection. Therefore, an enzyme-linked immuno-sorbent assay (ELISA) for MrgX2 was developed.

**Methods:**

Monoclonal and polyclonal MrgX2 specific antibodies were obtained from rabbits and mice immunized by MrgX2 peptides prepared. Indirect ELISA and Dot blot were used to determine antibody titers before a sandwich ELISA for MrgX2 was established. The whole blood from healthy subjects and CU patients was used to detect MrgX2 concentrations. The use of feasibility of this MrgX2-ELISA as a clinical detection tool was explored and diagnostic purposes was assessed.

**Results:**

The sandwich antibody ELISA method for MrgX2 was established with good linearity regression (R^2^ = 0.9910). The lowest detection limit was 3.125 ng/mL. The quantification limit was 6.25 ng/mL. The sandwich ELISA for MrgX2 have good stability and high specificity. The initial truncation value of MrgX2 was 60.91 ng/mL (95% confidence interval). The whole blood MrgX2 concentrations in CU patients (median 98.01 ± 4.317 ng/mL, n = 75) was significantly increased compared to healthy subjects (58.09 ± 1.418 ng/mL, n = 75), with significant difference (p < 0.0001) and higher accuracy of (AUC = 0.8795). Comprehensive the frequency analysis of MrgX2 expression in 75 CU patients reference frequency distribution and ROC curve analysis, determined the threshold for CU patients as 71.23 ng/mL, with 81.33% sensitivity and 90.67% specificity.

**Conclusion:**

MrgX2-ELISA provides a useful and convenient method for detecting MrgX2 in whole blood samples. The MrgX2-ELISA will help improve the understanding of the role of MrgX2 in regulating chronic urticaria.

## Introduction

Chronic urticaria (CU) is characterized by the presence of daily hives daily for at least 6 weeks [1]. Mast cells (MCs) and eosinophils colocalize in urticarial lesions in CU patients. Eosinophils release granules proteins in them (major basic protein, MBP and eosinophil peroxidase, EPO) that activate the Mas-related G-protein coupled receptor member X2 (MrgX2) and thus cause MCs degranulation [2, 3]. MrgX2 is not exclusively expressed on human mast cells but also on basophils and eosinophils and that it mediates degranulation [4]. When compared to healthy subjects, skin MCs from CU patients expressed MrgX2 at higher levels in CU patients. Therefore, MrgX2 might present a new target for CU treatment [2].

MrgX2, one of G-protein coupled receptor (GPCR), mainly expressed in skin MCs which express tryptase and chymase (MCTC) [2, 5, 6]. Previous studies reported mast cell respond to antimicrobial host defense peptides (HDPs), neuropeptides Substance P (SP), U.S. Food and Drug Administration (FDA)-approved cationic drugs and opioids via MrgX2 [7, 8]. MrgX2 on the cell membrane of human MCs directly mediates pseudo allergic reactions induced by drugs such as quinolone antibiotics and neuromuscular relaxants [9]. In addition, antifungal drugs, aminoglycosides and sulfonamides activated MCs via MrgX2 and triggered degranulation [10]. Drug-induced pseudo allergic reactions mediated by MrgX2 affecting the occurrence and development of allergic reactions [11]. The serum MrgX2 levels were significantly higher in asthma patients than in healthy subjects, so MrgX2 may also be as a potential biomarker for predicting treatment outcomes in allergic asthma [12]. Thus, it can be hypothesized that expression level of MrgX2 in patients might allow to establish personalized CU and allergic asthma therapy.

Currently, clinical detection methods of MrgX2 expression is under investigated. This study aimed to develop robust and reliable Enzyme-linked immuno-sorbent assay (ELISA) for MrgX2 in human whole blood samples. Therefore, we developed a sandwich ELISA assay for human MrgX2 in human whole blood samples. The data demonstrate that the concentration of MrgX2 is increased in whole blood samples of CU patients when compared to healthy subjects.

## Materials and methods

Chemical: 3,3′,5,5′-Tetramethylbenzidin (TMB) was from Suo Laibao Technology Co., Ltd (Beijing, China); Horseradish peroxidase (HRP) was from Sigma-Aldrich (Shanghai, China); Human mononuclear cell separation solution (product ID: 25171004) was from Dongfang Huahui Technology Co., Ltd (Shanghai, China);

 Mem-PER™ Plus Membrane Protein Extraction Kit (product ID: 89842) was from ThermoFisherScientific (Shanghai, China); Nitrocellulose filter membrane (NC membrane) was from Bio-Rad Laboratories, Inc (Shanghai, China);

 Protein-G column chromatography was from GE Healthcare (Shanghai, China); Horseradish peroxidase (HRP)-conjugated rabbit secondary antibody (Goat Anti-Rabbit IgG, HRP) was from Zhuangzhi Biotech, Xi’an, China; Horseradish peroxidase (HRP)-conjugated mouse secondary antibody (Rabbit Anti- Mouse IgG, HRP) was from Zhuangzhi Biotech, Xi’an, China;

MrgX2 standard protein (Recombinant Human GPCR MRGX2 protein, ab165129) was from Abcam (Shanghai, China);

 Washing solution (composed of PBS and Tween 20; homemade);

 Coating solution (composed of sodium carbonate and sodium bicarbonate; homemade); Blocking solution (composed of gelatin, sucrose and BSA; homemade);

 Diluents solution (consisting of PBS, Tween 20 and BSA; homemade); Stop solution (2 M H_2_SO_4_; homemade).

### Preparation of human MrgX2 peptide (T1) immunogen

The amino acid sequence of human MrgX2 was sourced from the GenBank database and DNAStar software predicted a strong antigenicity and high hydrophilicity for the MrgX2 peptide (T1). T1 was synthesized by the manual solid-phase Fmoc method, and purified by reversed-phase high-performance liquid chromatography (HPLC). Purity of the T1 was tested by analytical HPLC (Agela C18-10 × 250 mm, flow rate: 1 mL/min). The chemical structure of T1 was characterized by MALDI-TOF mass spectrometry. T1 was bound to the carrier protein keyhole limpet hemocyanin (KLH) by a cysteine residue added to the N-terminus of the T1 chain, and the glutaraldehyde method.

### Preparation of anti-human MrgX2 peptide (T1) antibodies

Animal experiments were conducted in accordance with the “Administrative Measures for Experimental Animals” (Ministry of Science and Technology) and as approved by the Animal Ethics Committee at Xi’an Jiaotong University, Xi’an, China (Permit Number: XJTU 2011-0045).

Anti-MrgX2 polyclonal antibodies (Pabs) were produced by immunizing rabbits (3 pairs) with human MrgX2 peptide (T1). The T1-KLH conjugate (4.2 mg/pair) was dissolved in Freund's complete adjuvant (1:1 volume ratio). Take subcutaneous sub-point injections, spray alcohol on the animal’s dorsal midline to avoid areas with immune swelling. One injection was divided into four injections and injected into 4 different points. Freund’s incomplete adjuvant booster injections were given every 2 weeks. After each booster injection, serum was collected from the ear vein, and antibody production was detected by Dot Blot method. Then the antiserum was purified by saturated ammonium sulfate precipitation and protein-G column chromatography, and then titrated by Dot Blot method.

Anti-MrgX2 monoclonal antibodies (Mabs) were produced by immunizing mice (10 pairs) with human MrgX2 peptide (T1). The T1-KLH (0.9 mg/pair) conjugate was dissolved in Freund’s complete adjuvant (1:1 volume ratio). Take subcutaneous sub-point injections, spray alcohol on the animal’s dorsal midline to avoid areas with immune swelling. One injection is divided into four injections and injected into 4 different points. Freund’s incomplete adjuvant booster injections were given every 2 weeks. After each booster injection, the serum was collected from the tail vein of mice, and antibody production was detected by indirect ELISA. After cell fusion, subcloning and antibody affinity purification (using saturated ammonium sulfate and protein columns), the purified monoclonal antibodies were obtained. Finally, indirect ELISA was used to determine the titer of purified antibodies.

1 mg of each antibody was labeled with biotin (Biotin labeling method) for ELISA detection. Dilute each antibody with 0.1 mol/L sodium bicarbonate buffer (pH 8.0) to 1 mg/mL. Alternately use 0.1 mol/L sodium bicarbonate buffer (PH 8.0) to fully dialyze the antibody. Using 1 mL DMSO to dissolve biotin succinimide Ester (NHSB) (1 mg), and add 120 μL of NHSB solution to 1 mL of antibody solution, keep stirring at room temperature for 2 h; add 9.6 μL of 1 mol/L NH_4_Cl (per 25 μg NHSB plus 1 μL), stir at room temperature for 10 min. The labeled antibodies were then diluted in 50% glycerol and stored at − 20 °C until further use.

### MrgX2 antibody titer by indirect ELISA

Screening of monoclonal antibody producing cell lines and antibody identification was performed by indirect ELISA (96-well plates). Aliquots of 100 μL of T1 were diluted in coating solution and added onto a microtiter plate (final concentration: 50 ng/well) and incubated at 4 °C overnight. The plate was then washed 3 × with 300 μL/well of washing solution (manual washing), before 200 μL/well of blocking solution and incubated at 37 °C for 2 h. The plate was washed 3 × and 100 μL/well of antibody (1:1000) diluted with diluents solution or 100 μL/well of cell supernatant was added and incubated at 37 °C for 1 h. The microtiter plate was washed 3 × with washing solution and 100 μL/well HRP-conjugated anti-rabbit secondary antibody (1:1000) or anti-mouse secondary antibody (1:1000) diluted with diluents solution were added, and incubated at 37 °C for 1 h. The plate was washed 3×, and 100 μL/well of TMB substrate was added at 37 °C for 5 min. The reaction was stopped by adding 50 μL/well 2 M H_2_SO_4_ and the absorbtion was determined at a wavelength of 450 nm (Flexstation 3, Meigu MolecularDevices, Shanghai, China).

### Dot Blot for antibody titer determination

Screening and identification of polyclonal antibodies was performed using the Dot Blot. 100 ng/well of T1 (100 μL/well) was added to the nitrocellulose filter membrane and incubated at 37 °C for 30 min. The nitrocellulose filter membrane was washed 5 × with washing solution (manual washing), and 1 mL/well coating solution was added containing a size of 1 cm^2^ and incubated for 1 h. Wash the nitrocellulose filter membrane 5 × with washing solution, and add 1 mL/well of the polyclonal antibodies dilution (1:1000) and incubate for 2 h. Wash the membrane 5 × with washing solution, add 1 mL/well of horseradish peroxidase (HRP)-conjugated rabbit secondary antibody (1:1000) and incubate for 1 h on the shaker. The nitrocellulose filter membrane was washed 5 × with washing solution, and finally the processed nitrocellulose filter membrane is developed in a dark room using developer and fixer in accordance with conventional development method.

### Establishment of double antibody sandwich MrgX2*-ELISA*

The optimal concentrations of Mab capture antibody and biotin-Pab detection antibody were determined by checkerboard titration and the reaction conditions for each step of the MrgX2-ELISA were optimized. The human MrgX2-ELISA was developed using the reagents described above. 100 μL/well of Mab (4 μg/mL, diluted with coating solution) was added to a microtiter plate (96-well plates) and incubated at 4 °C overnight. The coating solution was then removed and the plate was washed 3 × with 300 μL/well washing solution and the plate was pat dried. Then, 200 μL/well of blocking solution were added and incubated at 37 °C for 2 h. The plate was washed 3 × and the MrgX2 standard protein (0.02 μg/μL) was diluted to 0.5 μg/mL with dilution solution, 100 μL/well was added and incubated at 37 °C for 1 h. The plate was washed 3 × and 100 μL/well Biotin-Pabs (0.5 μg/mL, diluted with diluents solution), was added, and incubated at 37 °C for 30 min. The plate was washed 3 × before 100 μL/well of avidin-HRP (1:500 in diluents solution) was added and incubated at 37 °C for 30 min. The plate was washed 3 × and 100 μL/well of TMB was added and the color was developed for 15 min. The reaction was stopped by adding 50 μL/well of stop solution and the absorbtion was determined at a wavelength of 450 nm (Flexstation 3, Meigu MolecularDevices, Shanghai, China).

### Optimizing the sandwich MrgX2*-ELISA to blood samples*

The above described sandwich MrgX2-ELISA was used to compute the standard curve, detection limit, limit of quantification, inter-assay precision, intra-assay precision, accuracy, stability and specificity. The stability of the MrgX2-ELISA was tested after 7 days storage at 37 °C. The sensitivity of the MrgX2-ELISA was calculated using the guidelines provided by the National Committee for Clinical Laboratory Standards (NCCLS) evaluation protocol [13].

### Study design of the CU trial

This study was registered at the Chinese Clinical Trail Registry (# ChiCTR1900025723), the ethical approval was given by Ethics Committee at Xi’an Jiaotong University (Xi’an, China). All specimens in this study were obtained after individual signed informed consent of each participant.

This study design was a single-center, random sampling, case–control study. Human whole blood samples of CU patients (n = 75, age 10–70 years, median age 40 years) were obtained from the Department of Dermatology in the Second Affiliated Hospital of Xi’an Jiaotong University (Xi’an, China), and human whole blood samples of control group (n = 75, age 18–76 years, median age 47 years) were sourced from the Department of Physical Examination in the Second Affiliated Hospital of Xi’an Jiaotong University (Xi’an, China).

### Study subjects

Inclusion criteria:

 The principle of case and control was applied to sample collection.

Sample collection criteria for CU patients:

1. CU was diagnosed as: at least three episodes of symptoms such as weekly wheals and pruritus over a period of more than 6 weeks.
2. Age 6–80 years.
3. No recent history of other diseases except CU.
4. Not pregnant.

Sample collection criteria for control group:

1. No history of CU.
2. No recent history of other diseases.
3. No family history of CU.
4. Age 6–80 years.
5. Not pregnant.

Exclusion criteria:

 Patients who had at least one of the following indicators were excluded from the study:

1. Unclear symptoms, and inability to confirm CU.
2. Patients with confirmed physical urticaria.
3. Patients who received systemic treatment of anti-histamines or glucocorticoids within 2 weeks prior to sample collection.

### *Clinical application of the sandwich* MrgX2*-ELISA*

Leukocytes were isolated and purified from fresh whole blood samples using human mononuclear cell separation solution in a final volume of 1 mL according to the instructions. Mem-PER™ Plus Membrane Protein Extraction Kit was used to lyse the cells and extract leukocytes membrane proteins according to the instructions. The MrgX2-ELISA method was used to detect the expression of MrgX2 protein in leukocytes protein extracts from CU patients (n = 75) and healthy subjects (n = 75).

The expression levels of MrgX2 protein in the whole blood (1 mL whole blood) of CU patients and healthy subjects were compared. The frequency distribution data of 75 healthy subjects was determined, the cutoff value of CU patients was derived, and the ROC curve was constructed based on the results of CU patients and healthy subjects.

### Data analysis

GraphPad Prism 7 software (GraphPad Software, San Diego, CA, USA) was used for ELISA standard curves. For each group, the median, 25th percentile, 75th percentile, and interquartile range of MrgX2 were determined. Two-tailed unpaired Student’s t-test was applied by GraphPad Prism 7 software, and* p* value of < 0.05 was considered as statistically significant.

## Results

### Preparation of human MrgX2 peptide (T1) immunogen

**Fig. 1:**
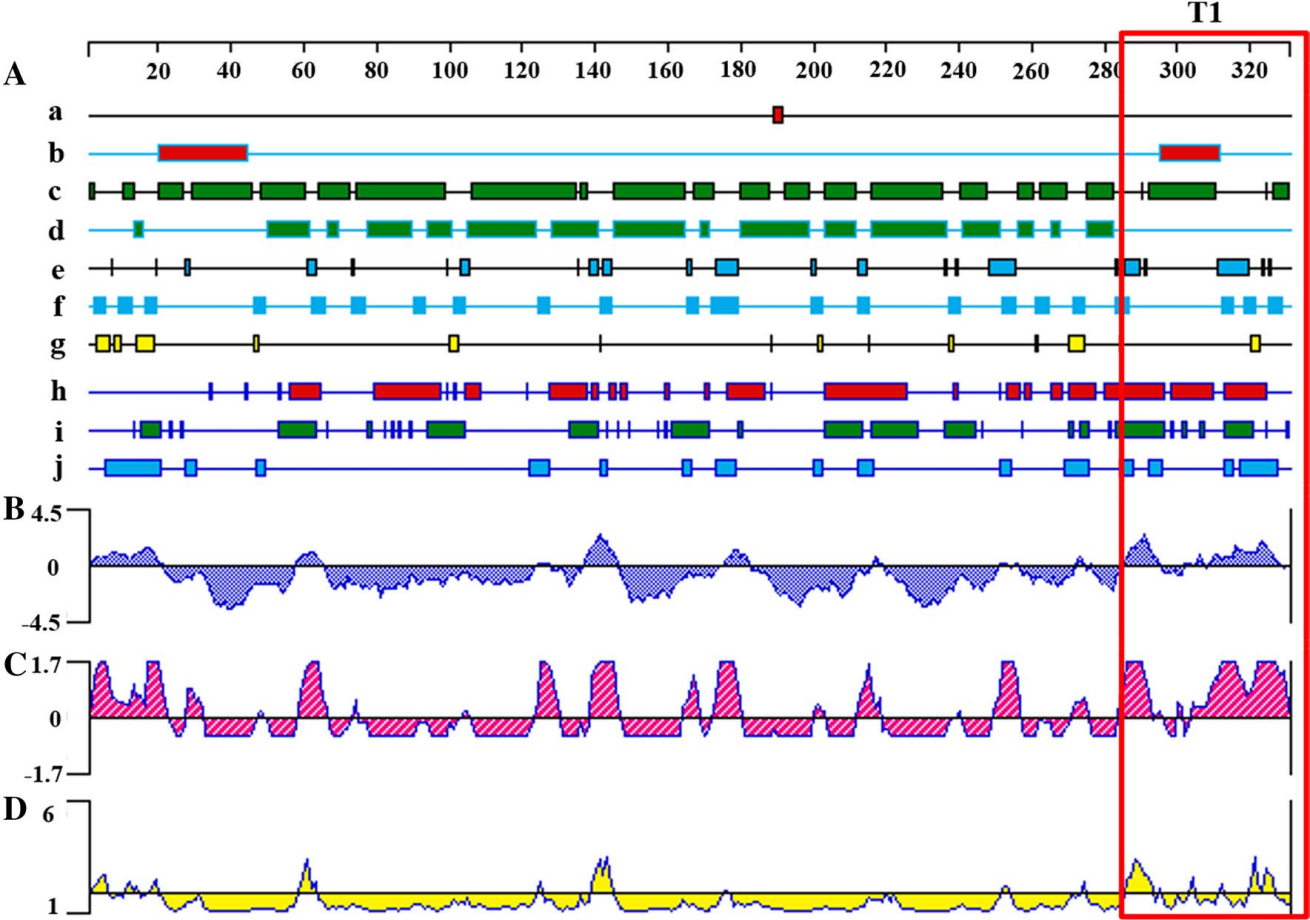
Prediction of immunogenicity of human MrgX2 antigen by bioinformatics method. **A** Prediction of the secondary structure of the human MrgX2 antigen (**a**) Prediction of the alpha helix of the sequence by the Gamier-Robson method. **b** Prediction of the alpha helix of the sequence by the Chou-Fasman method. **c** Prediction of the beta fold of the sequence by the Gamier-Robson method. **d** The Chou-Fasman method predicts the β-fold of the sequence. **e** The Gamier-Robson method predicts the rotation angle of the sequence. **f** The Chou-Fasman method predicts the rotation angle of the sequence. **g** The Gamier-Robson method predicts the sequence curl. **h** Eisenberg method predicts alpha-helix hydrophilicity. **i** Eisenberg method predicts beta-sheet hydrophilicity. **j** Karplus-Schulz method predicts sequence flexibility. **B** Kyte-Doolittle method predicts sequence hydrophilicity. **C** Jameson-Wolf method predicts sequence antigen index. **D** Emini method predicts sequence surface accessibility. T1 in the red box showed the 286–330 amino acid sequence of human MrgX2 peptide

**Fig. 2:**
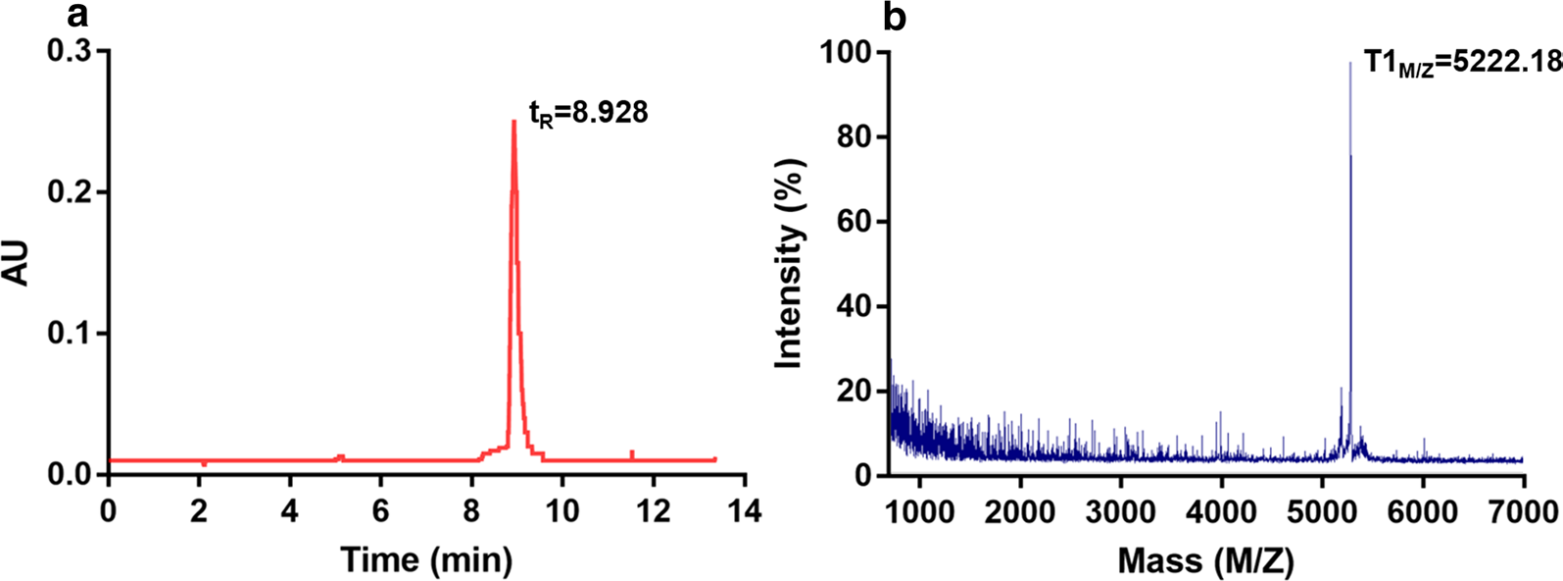
Purity and chemical structure characterization of human MrgX2 peptide (T1). **a** HPLC analysis of T1 (t_R_ = 8.928 min). **b** MALDI-TOF spectrum of the T1 (T1_M/Z_ = 5222.18 Da)

The results of evaluation of the two-dimensional structure of human MrgX2 protein are shown in Fig. 1. Based on the considerations of immunogenicity, antigenicity, and hydrophilicity, the MrgX2 peptide (T1) was selected as the most classic functional domain of human MrgX2 protein that is most likely to form cell epitopes. Thus, the T1 was synthesized and purified. Analytical high-performance liquid chromatography confirmed the purity (> 90%) of the synthesized peptide (Fig. 2a), which meets the requirements of animal immunity. The structure of T1 was characterized by MALDI-TOF mass spectrometry and the molecular weight of the T1 was determined to be 5222.18 Da (Fig. 2b), which is equivalent to the amino acid size of the peptide sequence.

### Identification of human MrgX2 antibodies

**Fig. 3:**
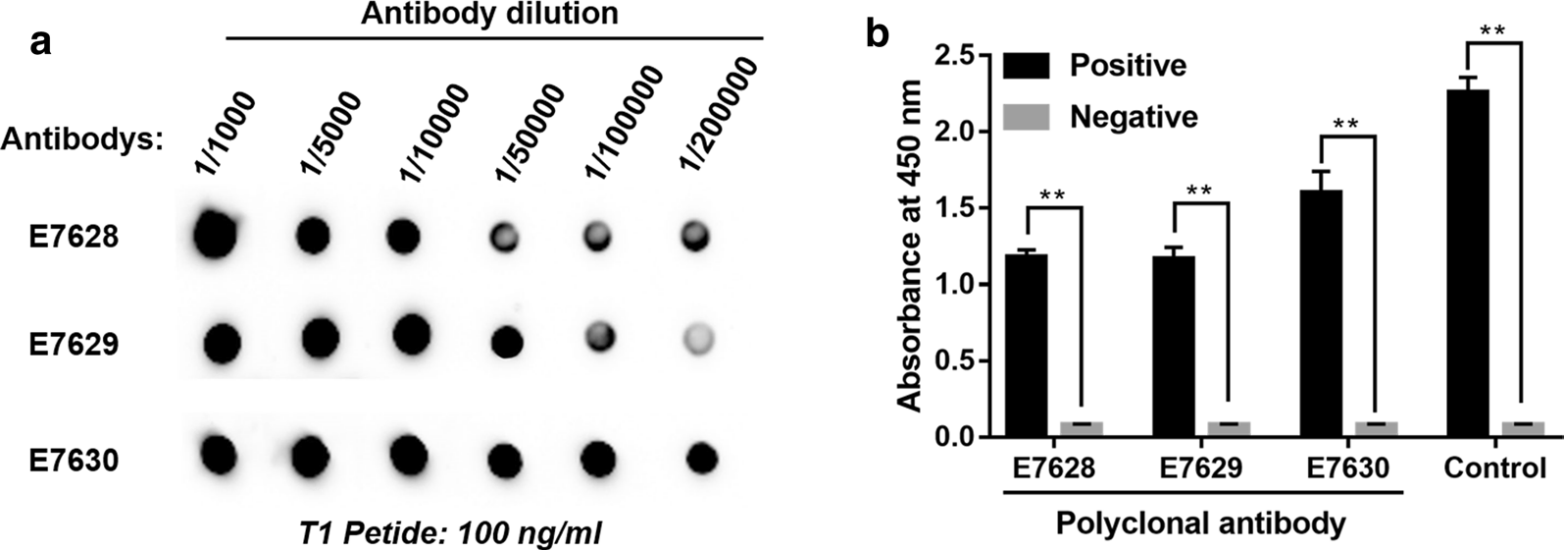
Titer detection diagram of human MrgX2 rabbit polyclonal antibody. **a** Dot blot method to verify the potency of human MrgX2 rabbit polyclonal purified antibody recognition polypeptide. **b** Indirect ELISA method to verify the ability of human MrgX2 rabbit polyclonal purified antibody to recognize natural MrgX2 protein. Student’s t test (nonparametric tests) was used to determine statistical significance. Data are expressed as mean ± SEM from at least three independent experiments. **p < .01, vs negative control

**Fig. 4:**
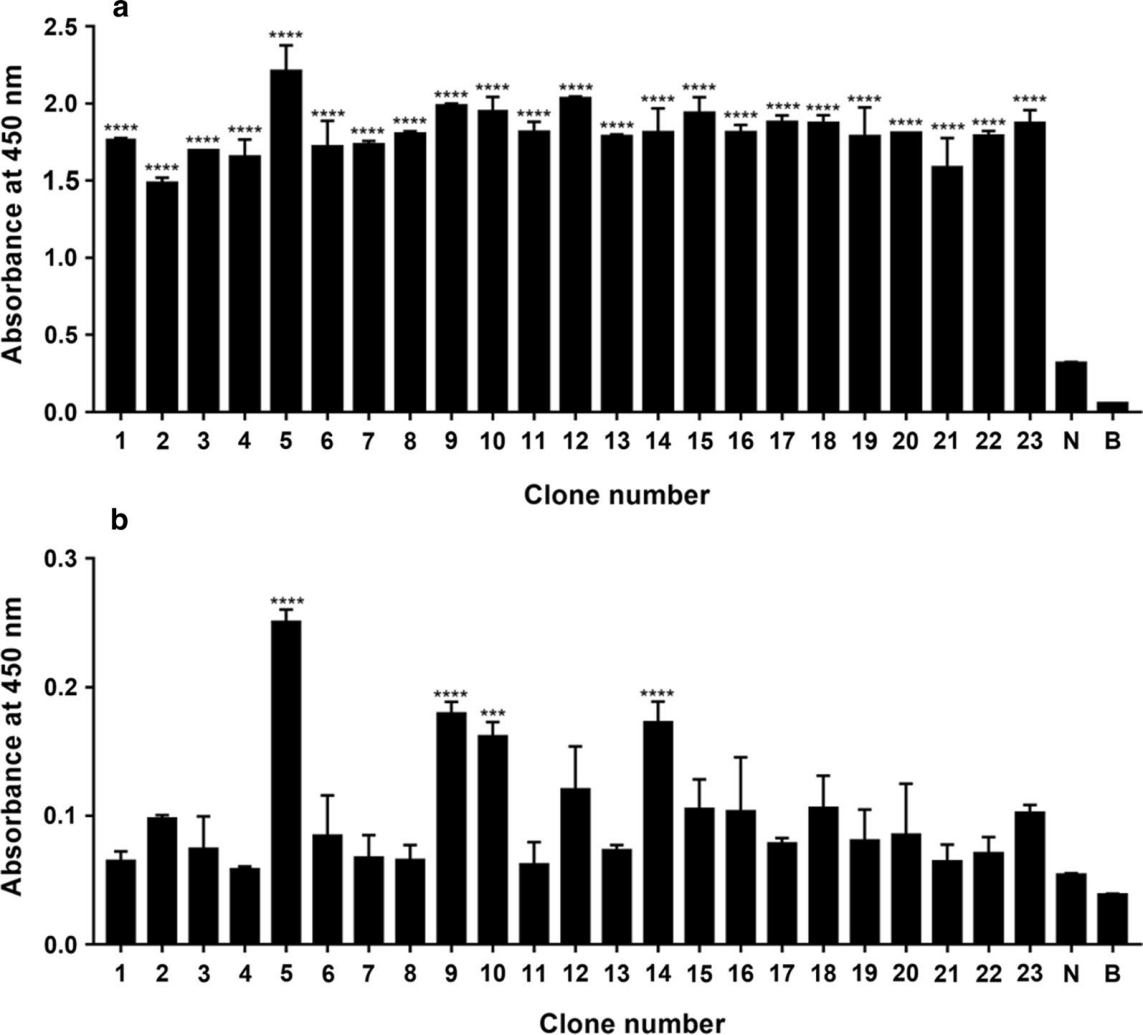
Figure of titer detection of human MrgX2 mouse monoclonal antibody (1–23: Monoclonal cell line, N: Negative, B: Blank). **a** Indirect ELISA method to examine the ability of the supernatant antibody of human MrgX2 mouse monoclonal cell line to recognize T1. **b** Indirect ELISA method to examine the ability of the supernatant antibody of human MrgX2 mouse monoclonal cell line to recognize natural MrgX2 protein. One‑way analysis of variance (Bonferroni’s multiple comparisons test) was used to determine statistical significance. Data are expressed as mean ± SEM from at least three independent experiments. ***p < .001, ****p < .0001 vs negative control

Dot blot analysis confirmed that Pabs concentration in serum of the animals was high, the maximum titer of antiserum can reach 1:200,000. (Fig. 3a). The ability of Pabs to specifically recognize natural human MrgX2 protein was verified using indirect ELISA method (Fig. 3b). Indirect ELISA was also used to confirm the concentration of Mabs in the mouse monoclonal cell line (Fig. 4a) and that it could specifically recognize the natural human MrgX2 protein (Fig. 4b).

### Establishment of human MrgX2-ELISA

**Fig. 5:**
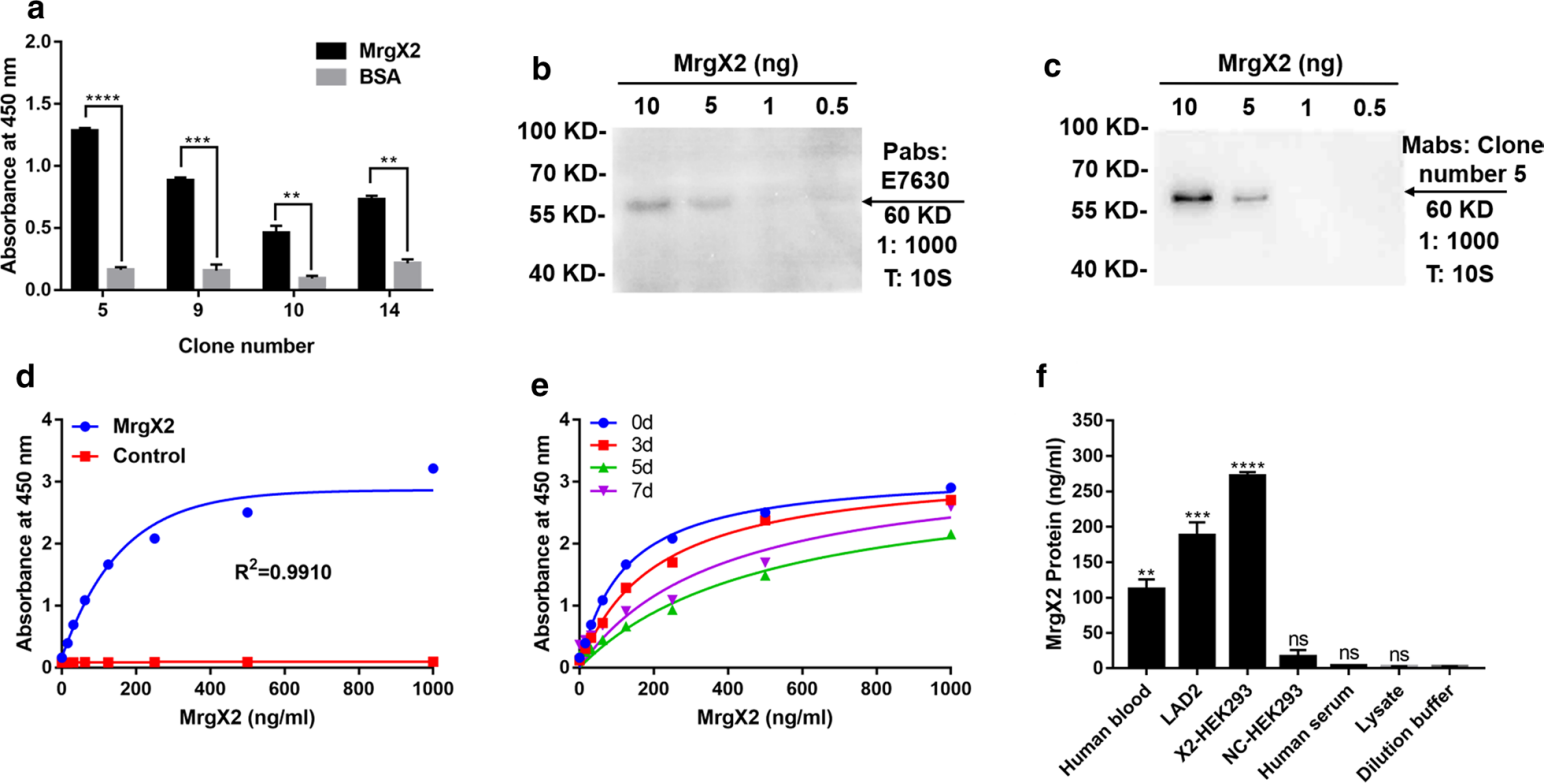
Establishment and methodological investigation of human MrgX2-ELISA. **a** Double antibody sandwich screening of matched antibodies. Student’s t test (nonparametric tests) was used to determine statistical significance. **b** Western blot to examine the specificity of E7630 rabbit polyclonal purified antibody. **c** Western blot to investigate the specificity of mouse monoclonal purified antibody. **d** Human MrgX2-Inspection of standard curve of ELISA. **e** Investigation of stability of human MrgX2-ELISA. **f** Investigation of specificity of human MrgX2-ELISA. One-way analysis of variance (Bonferroni’s multiple comparisons test) was used to determine statistical significance. Data are expressed as mean ± SEM from at least three independent experiments. **p < .01, ***p < .001, ****p < .0001 *vs.* negative control

Orthogonal experiments were used to screen for the best double-antibody sandwich paired antibodies. Monoclonal antibody was used as the capture antibody and biotin-Pabs was used as the detection antibody (Fig. 5a). Immunoblotting was used to analyze the specific recognition ability of Pabs to the natural MrgX2 protein. As shown in Fig. 5b, natural human MrgX2 is a single peptide of about 60 kDa, suggesting that rabbit polyclonal antibodies can recognize natural human antigens. Furthermore, as shown in Fig. 5c, Mabs can also recognize natural human MrgX2.

A sandwich ELISA method was employed to detect MrgX2 in human whole blood samples. Plotting the standard dose–response curve of human MrgX2 protein on a scale of 0–1000 ng/mL revealed that the correlation coefficient R^2^ = 0.9910 (Fig. 5d). The detection limit was 3.125 ng/mL and the limit of quantification was 6.25 ng/mL (Additional file: 7 Table S1). By measuring the recovery from the solution containing three added doses of human MrgX2 fusion protein, the intra-batch coefficient of variation (CV) was found to be less than 11.88% (Additional file: 7 Table S2). The inter-batch coefficient of variation (CV) was found to be less than 9.163% (Additional file: 7 Table S2). Although the standard curve was found to have a slight downward trend through accelerated experiments, it had a good linearity (Fig. 5e). The specificity of the MrgX2-ELISA was investigated by testing human serum, human plasma, LAD2 cell membrane protein, the highly expressed cell membrane protein X2-HEK293, the cell membrane protein NC-HEK293, and protein lysate (Fig. 5f). The sensitivity of the MrgX2-ELISA method was calculated to be 7.75 ng/mL (Additional file: 7 Table S1). Therefore, the reliability of the newly established ELISA method was evaluated to be of high precision.

### Human MrgX2-ELISA for detection of CU patients

**Fig. 6:**
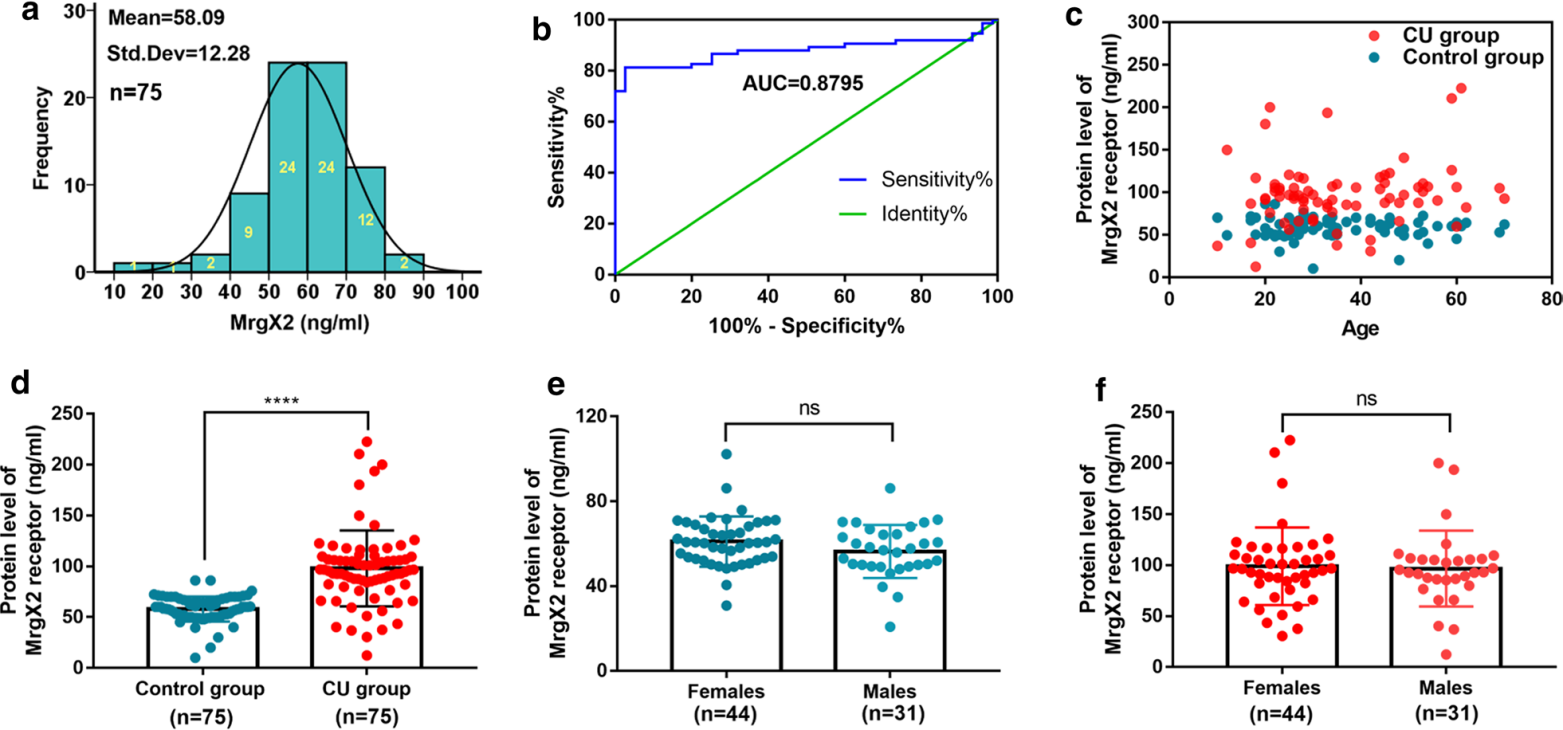
Clinical application of human MrgX2-ELISA. **a** Healthy subjects frequency distribution of human blood MrgX2 concentration in the population (n = 75). **b** ROC curve of human MrgX2 protein expression (n = 150), green line represents the diagnostic reference line; blue line represents the ROC curve of MrgX2. **c** Scatter plot of blood MrgX2 concentration in CU patients (n = 75) and healthy subjects (n = 75). **d** Histogram of blood MrgX2 concentration in CU patients (n = 75) and healthy subjects (n = 75). **e** Comparison of blood MrgX2 concentration in healthy male (n = 31) and healthy famale (n = 44). **f** Comparison of blood MrgX2 concentration in CU male (n = 31) and CU famale (n = 44). Student’s *t* test (nonparametric tests) was used to determine statistical significance. Data are expressed as mean ± SEM from at least three independent experiments. ****p < .0001 vs. control group

Using the established MrgX2-ELISA method, we determined the cut-off value and normal detection range of MrgX2 in human whole blood samples. Based on the frequency distribution data of 75 healthy subjects (Fig. 6a), the initial cut-off value of MrgX2 was 60.91 ng/mL (95% confidence interval). The sandwich method was used to evaluate MrgX2 in 150 clinical samples with 75 CU patients and 75 healthy subjects. Based on the results from these samples, the area under the ROC curve was determined to be 0.8795 (Fig. 6b). When the threshold was 75.88 ng/mL, the sensitivity of the kit was 81.33% and the specificity was 96%. Based on the reference frequency distribution and ROC curve, the threshold was determined to be 71.23 ng/mL, the reasonable sensitivity was 81.33%, and the specificity was 90.67%. The level of MrgX2 (98.01 ± 4.317 ng/mL) in the blood of CU patients was significantly higher than that of healthy subjects (58.09 ± 1.418 ng/mL, *p* < 0.0001) (Fig. 6c, d) (Additional file: 7 Table S3). The concentration of MrgX2 in healthy female (median 61.01 ± 1.784, n = 44) was not significantly different from healthy male (median 56.35 ± 2.238, n = 31) (Fig. 6e, Additional file: 7 Table S4); the concentration of MrgX2 in female CU patients (median 98.99 ± 5.723 ng/mL, n = 44) was also not significantly different from male CU patients (median 96.63 ± 6.669 ng/mL, n = 31) (Fig. 6f, Additional file: 7 Table S4).

## Discussion

Our results demonstrated that the MrgX2-ELISA method described here is capable of measuring MrgX2 concentration in human whole blood samples. Currently, no effective clinical detection methods exist for MrgX2.

Our MrgX2-ELISA method has the inherent advantage of the dual antibody sandwich detection. In particular, the response intensity is directly related to an increase in MrgX2 concentration. Since two specific antibodies against MrgX2 protein are used, the detection results are accurate and reliable [14]. From a practical viewpoint, ELISA can be performed in clinical laboratories and test results can be obtained within three hours without the need for complex equipment or highly specialized operator expertise. Another advantage of our MrgX2-ELISA method is its limit of quantification at 3.125 ng/mL. This is especially important, since we observed that whole blood MrgX2 concentrations of healthy subjects were < 10 ng/mL.

From a practical standpoint, the advantage of ELISA method over Liquid chromatography-mass spectrometer (LC–MS) is that ELISA method can be performed in clinical laboratories that do not have the complex equipment or the highly specialized operator expertise required to perform LC–MS type assays. In addition, unlike LC–MS, ELISA also has the potential for higher throughput and therefore provides the basis for first dual antibody sandwich immunoassay to measure MrgX2. Our results indicate that we have successfully established a dual-antibody sandwich MrgX2-ELISA detection method with high specificity, accuracy, reproducibility and sufficient sensitivity that can be used for detection MrgX2 in human whole blood samples.

It is well known that while mast cells are located around tissues, and leukocytes are distributed in peripheral blood [15], their common feature is the release of allergic mediators such as histamine through the degranulation pathway. These are the key effector cells that trigger IgE-mediated type I allergic reactions [16]. Mast cells and basophils are derived from bone marrow differentiation and have similar biological characteristics [17]. For monitoring allergic diseases, blood basophils can reflect the situation in the body as comprehensively as possible [18]. The sandwich ELISA method described here can be used clinically to further increase our understanding of the role of MrgX2 in regulating chronic urticaria.

This high-throughput method is particularly important for clinical trials to determine the concentration of MrgX2 protein in human whole blood samples. Based on the frequency distribution data and ROC curves of 75 healthy subjects, we determined the initial truncation value of MrgX2 to be 60.91 ng/mL (95% confidence interval). Using the established MrgX2-ELISA method, the human whole blood MrgX2 concentrations were found to be higher in CU patients than in healthy subjects. The results were similar in the skin MCs that express MrgX2 at higher levels in CU patients than in healthy subjects [2]. Furthermore, there was no significant difference in the MrgX2 protein expression in male and female CU patients. However, it should be noted that owing to the limited number of patients in our study, data obtained from CU patients must be interpreted with caution.

In addition to these observations, the dual antibody sandwich MrgX2-ELISA method has several other uses. Allergic asthma, the most common phenotype of asthma, is clinically defined by the presence of allergic sensitization and a correlation between asthma symptoms and allergen exposure [19, 20]. MrgX2 may promote the development of asthma and may serve as a potential new target for regulating this chronic inflammatory disease [21]. MrgX2 may also be as a potential biomarker for predicting treatment outcomes in allergic asthma [12]. Mast cells are important effector cells that orchestrate the development of airway hyperresponsiveness and inflammation in asthma via their close interaction with smooth muscle cells, T cells and leukocytes in the airway [22–24]. For asthma patients, the MrgX2-ELISA test results may indicate whether MrgX2 levels are correlated with the disease and provide richer clinical data for clinical diagnosis and treatment. MrgX2 receptors also play an important role in pruritus and erythema-related inflammatory disorders. Our MrgX2-ELISA method can be used to determine the human whole blood MrgX2 concentrations to better guide the treatment of other MrgX2 related chronic inflammatory diseases.

In summary, our MrgX2-ELISA method can help improve our understanding of the role of MrgX2 in regulating chronic urticaria. The use of the two antibodies in the sandwich format provides specificity for the active form of the protein, with a limit of quantification of 3.125 ng/mL, and a broad dynamic range for the clinical detection of MrgX2 related chronic urticaria.

## Conclusion

MrgX2-ELISA provides a useful and convenient method for detecting MrgX2 in human whole blood. Our method could be a rewarding test as currently there is no commercially available in vitro tests to neither diagnose urticaria nor to follow up disease activity. This method provides guidance and reference values for the development of MrgX2 immunoassay and for the clinical detection of other MrgX2 related allergic diseases.

## Supplementary information

**Additional file 1: Fig. S1.** Prediction of immunogenicity of human MrgX2 antigen by bioinformatics method. **A** Prediction of the secondary structure of the human MrgX2 antigen **a** Prediction of the alpha helix of the sequence by the Gamier-Robson method. **b** Prediction of the alpha helix of the sequence by the Chou-Fasman method. **c** Prediction of the beta fold of the sequence by the Gamier-Robson method. **d** The Chou-Fasman method predicts the β-fold of the sequence. **e** The Gamier-Robson method predicts the rotation angle of the sequence. **f** The Chou-Fasman method predicts the rotation angle of the sequence. **g** The Gamier-Robson method predicts the sequence curl. **h** Eisenberg method predicts alpha-helix hydrophilicity. **i** Eisenberg method predicts beta-sheet hydrophilicity. **j** Karplus-Schulz method predicts sequence flexibility. **B** Kyte-Doolittle method predicts sequence hydrophilicity. **C** Jameson-Wolf method predicts sequence antigen index. **D** Emini method predicts sequence surface accessibility. T1 in the red box showed the 286–330 amino acid sequence of human MrgX2 peptide.

**Additional file 2: Fig. S2.** Purity and chemical structure characterization of human MrgX2 peptide (T1). **a** HPLC analysis of T1 (tR = 8.928 min). **b** MALDI-TOF spectrum of the T1 (T1M/Z = 5222.18 Da).

**Additional file 3: Fig. S3.** Titer detection diagram of human MrgX2 rabbit polyclonal antibody. **a** Dot blot method to verify the potency of human MrgX2 rabbit polyclonal purified antibody recognition polypeptide. **b** Indirect ELISA method to verify the ability of human MrgX2 rabbit polyclonal purified antibody to recognize natural MrgX2 protein. Student’s t test (nonparametric tests) was used to determine statistical significance. Data are expressed as mean ± SEM from at least three independent experiments. **p < .01, vs negative control.

**Additional file 4: Fig. S4.** Figure of titer detection of human MrgX2 mouse monoclonal antibody (1–23: Monoclonal cell line, N: Negative, B: Blank). **a** Indirect ELISA method to examine the ability of the supernatant antibody of human MrgX2 mouse monoclonal cell line to recognize T1. **b** Indirect ELISA method to examine the ability of the supernatant antibody of human MrgX2 mouse monoclonal cell line to recognize natural MrgX2 protein. One way analysis of variance (Bonferroni’s multiple comparisons test) was used to determine statistical significance. Data are expressed as mean ± SEM from at least three independent experiments. ***p < .001, ****p < .0001 vs negative control.

**Additional file 5: Fig. S5.** Establishment and methodological investigation of human MrgX2-ELISA. **a** Double antibody sandwich screening of matched antibodies. Student’s t test (nonparametric tests) was used to determine statistical significance. **b** Western blot to examine the specificity of E7630 rabbit polyclonal purified antibody. **c** Western blot to investigate the specificity of mouse monoclonal purified antibody. **d** Human MrgX2-Inspection of standard curve of ELISA. **e** Investigation of stability of human MrgX2-ELISA. **f** Investigation of specificity of human MrgX2-ELISA. One-way analysis of variance (Bonferroni’s multiple comparisons test) was used to determine statistical significance. Data are expressed as mean ± SEM from at least three independent experiments. **p < .01, ***p < .001, ****p < .0001 vs. negative control.

**Additional file 6: Fig. S6.** Clinical application of human MrgX2-ELISA. **a** Healthy subjects frequency distribution of human blood MrgX2 concentration in the population (n = 75). **b** ROC curve of human MrgX2 protein expression (n = 150), green line represents the diagnostic reference line; blue line represents the ROC curve of MrgX2. **c** Scatter plot of blood MrgX2 concentration in CU patients (n = 75) and healthy subjects (n = 75). **d** Histogram of blood MrgX2 concentration in CU patients (n = 75) and healthy subjects (n = 75). **e** Comparison of blood MrgX2 concentration in healthy male (n = 31) and healthy famale (n = 44). **f** Comparison of blood MrgX2 concentration in CU male (n = 31) and CU female (n = 44). Student’s t test (nonparametric tests) was used to determine statistical significance. Data are expressed as mean ± SEM from at least three independent experiments. ****p < .0001 vs. control group.

**Additional file 7: Table S1** Detection line, quantitative limit, linear range and accuracy. **Table S2** Inter-Assay and Intra-Assay Precision. **Table S3** MrgX2 concentrations for groups of healthy subjects and CU patients. **Table S4** Comparisons of MrgX2 concentrations between respective groups of healthy subjects and CU patients.

## Data Availability

The datasets used and/or analysed during the current study are available from the corresponding author on reasonable request.

## Abbreviations

CU: Chronic urticaria
CV: Coefficient of variation
EPO: Eosinophil peroxidase
HDPs: Host defense peptides
GPCR: G-protein coupled receptor
HPLC: High-performance liquid chromatography
HRP: Horseradish peroxidase
KLH: Keyhole limpet hemocyanin
LAD2: Laboratory of allergic disease 2
LC–MS: Liquid chromatography–mass spectrometer
Mabs: Monoclonal antibodies
MBP: Major basic protein
MCT: Mast cells tryptase
MCTC: Mast cells tryptase and chymase
MrgX2: Mas-related G-protein coupled receptor member X2
Pabs: Polyclonal antibodies
SP: Substance P
TMB: 3,3′,5,5′-Tetramethylbenzidine
FDA: U.S. Food and Drug Administration
