# Supplementary figures and images for: A novel ELISA method to determine human MrgX2 in chronic urticaria
Source: Clin Transl Allergy. 2020 Dec 9;10:61. doi: 10.1186/s13601-020-00361-8 (PMC7727259; doi:10.1186/s13601-020-00361-8)

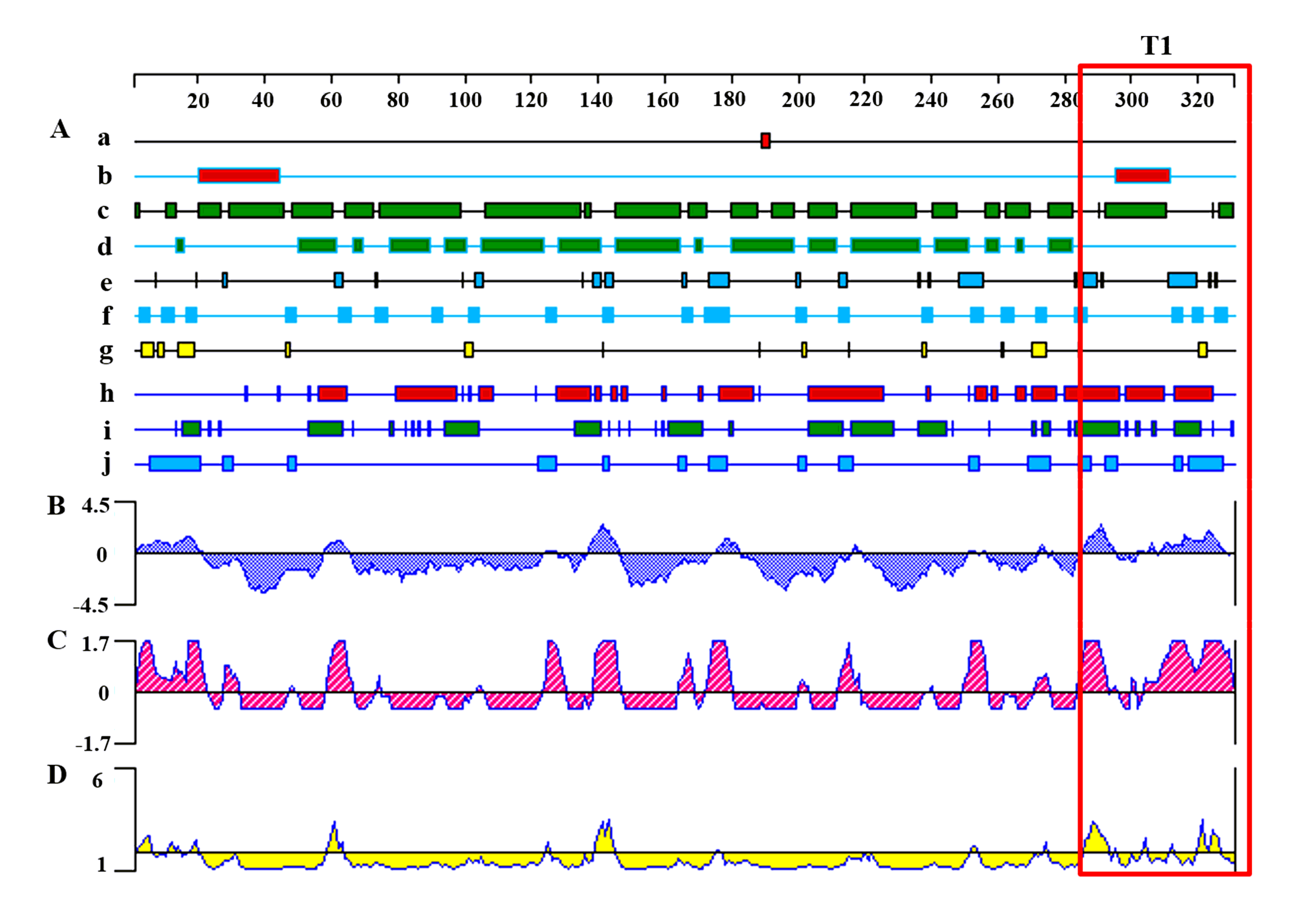

Supplement: Supplementary file 1 — Additional file 1: Fig. S1. Prediction of immunogenicity of human MrgX2 antigen by bioinformatics method. A Prediction of the secondary structure of the human MrgX2 antigen a Prediction of the alpha helix of the sequence by the Gamier-Robson method. b Prediction of the alpha helix of the sequence by the Chou-Fasman method. c Prediction of the beta fold of the sequence by the Gamier-Robson method. d The Chou-Fasman method predicts the β-fold of the sequence. e The Gamier-Robson method predicts the rotation angle of the sequence. f The Chou-Fasman method predicts the rotation angle of the sequence. g The Gamier-Robson method predicts the sequence curl. h Eisenberg method predicts alpha-helix hydrophilicity. i Eisenberg method predicts beta-sheet hydrophilicity. j Karplus-Schulz method predicts sequence flexibility. B Kyte-Doolittle method predicts sequence hydrophilicity. C Jameson-Wolf method predicts sequence antigen index. D Emini method predicts sequence surface accessibility. T1 in the red box showed the 286–330 amino acid sequence of human MrgX2 peptide. [file 13601_2020_361_MOESM1_ESM.png]

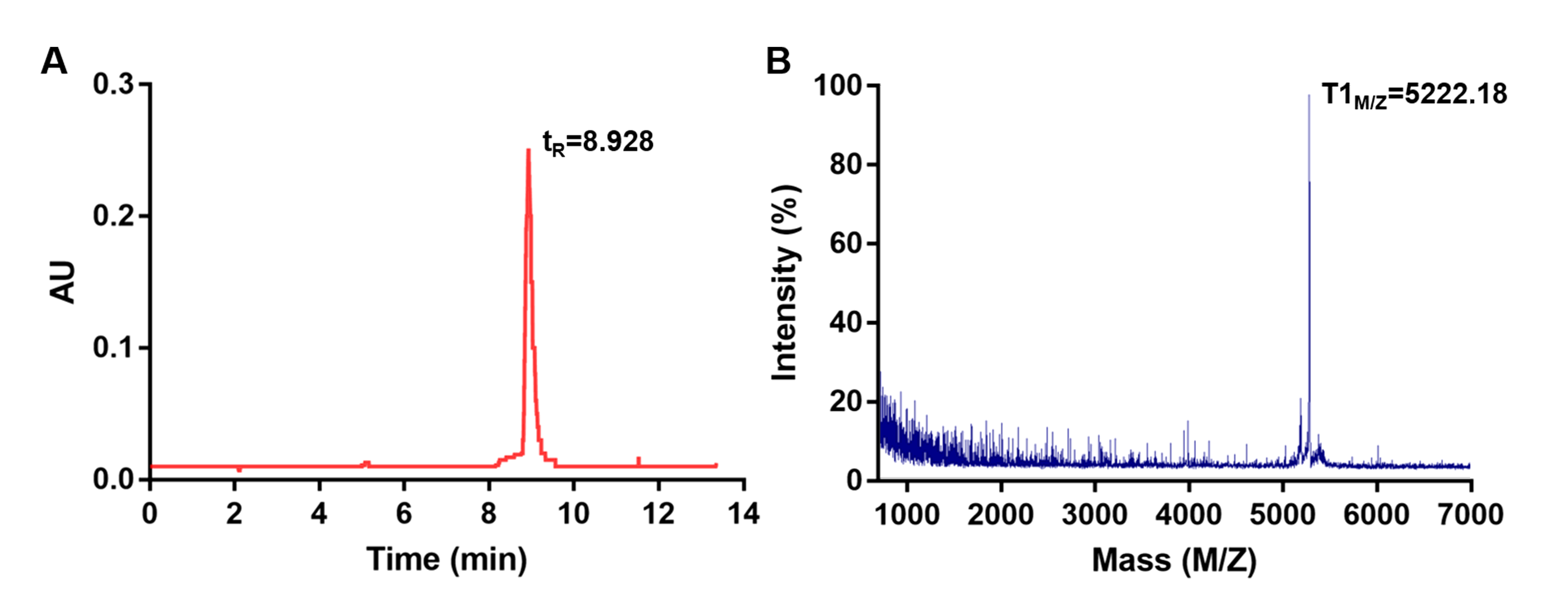

Supplement: Supplementary file 2 — Additional file 2: Fig. S2. Purity and chemical structure characterization of human MrgX2 peptide (T1). a HPLC analysis of T1 (tR = 8.928 min). b MALDI-TOF spectrum of the T1 (T1M/Z = 5222.18 Da). [file 13601_2020_361_MOESM2_ESM.png]

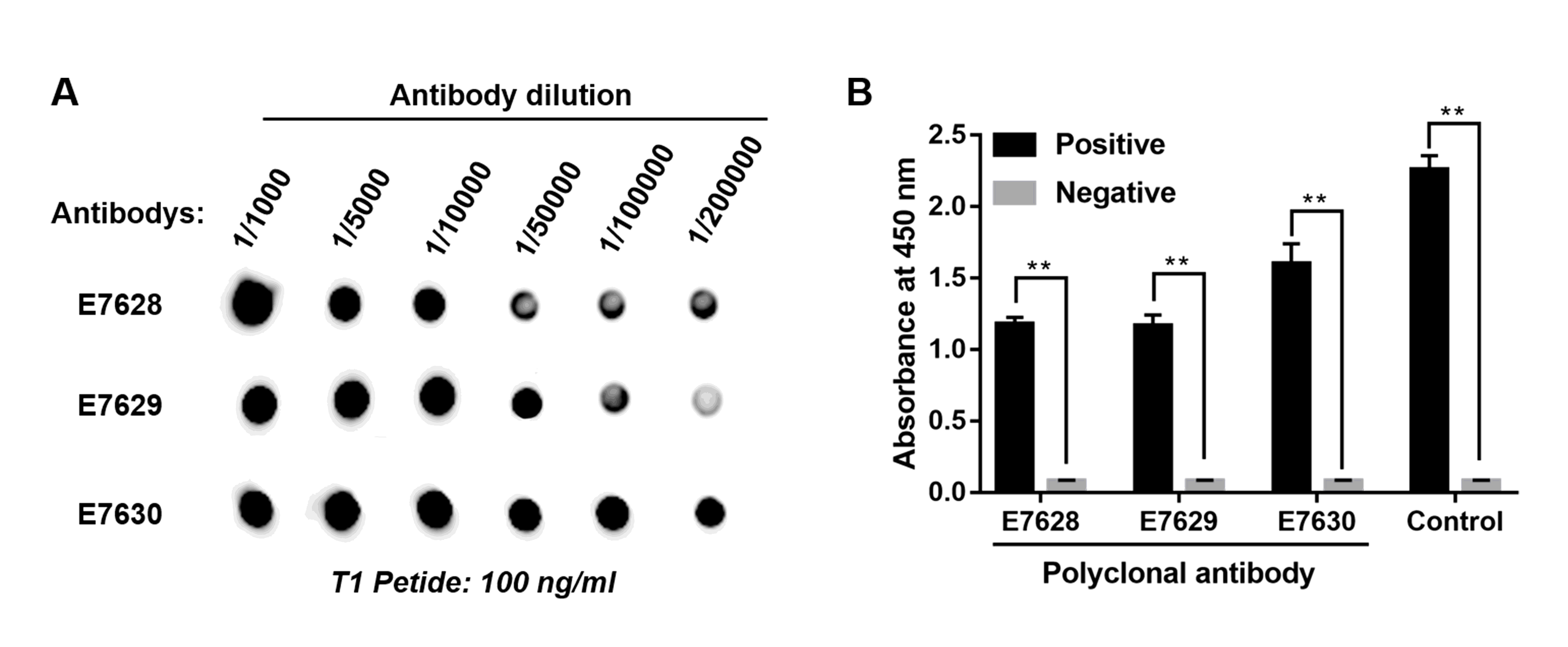

Supplement: Supplementary file 3 — Additional file 3: Fig. S3. Titer detection diagram of human MrgX2 rabbit polyclonal antibody. a Dot blot method to verify the potency of human MrgX2 rabbit polyclonal purified antibody recognition polypeptide. b Indirect ELISA method to verify the ability of human MrgX2 rabbit polyclonal purified antibody to recognize natural MrgX2 protein. Student’s t test (nonparametric tests) was used to determine statistical significance. Data are expressed as mean ± SEM from at least three independent experiments. **p < .01, vs negative control. [file 13601_2020_361_MOESM3_ESM.png]

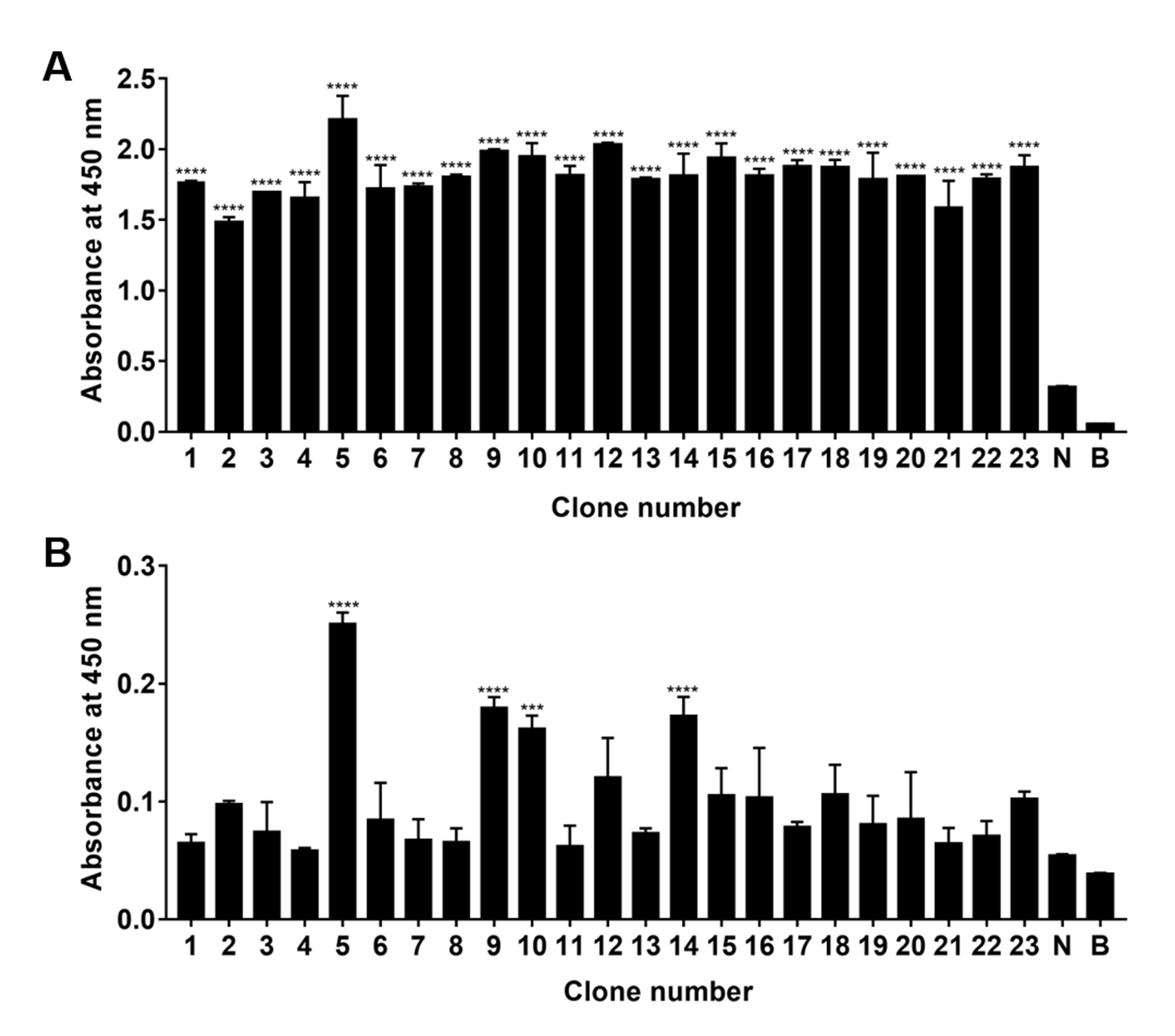

Supplement: Supplementary file 4 — Additional file 4: Fig. S4. Figure of titer detection of human MrgX2 mouse monoclonal antibody (1–23: Monoclonal cell line, N: Negative, B: Blank). a Indirect ELISA method to examine the ability of the supernatant antibody of human MrgX2 mouse monoclonal cell line to recognize T1. b Indirect ELISA method to examine the ability of the supernatant antibody of human MrgX2 mouse monoclonal cell line to recognize natural MrgX2 protein. One way analysis of variance (Bonferroni’s multiple comparisons test) was used to determine statistical significance. Data are expressed as mean ± SEM from at least three independent experiments. ***p < .001, ****p < .0001 vs negative control. [file 13601_2020_361_MOESM4_ESM.png]

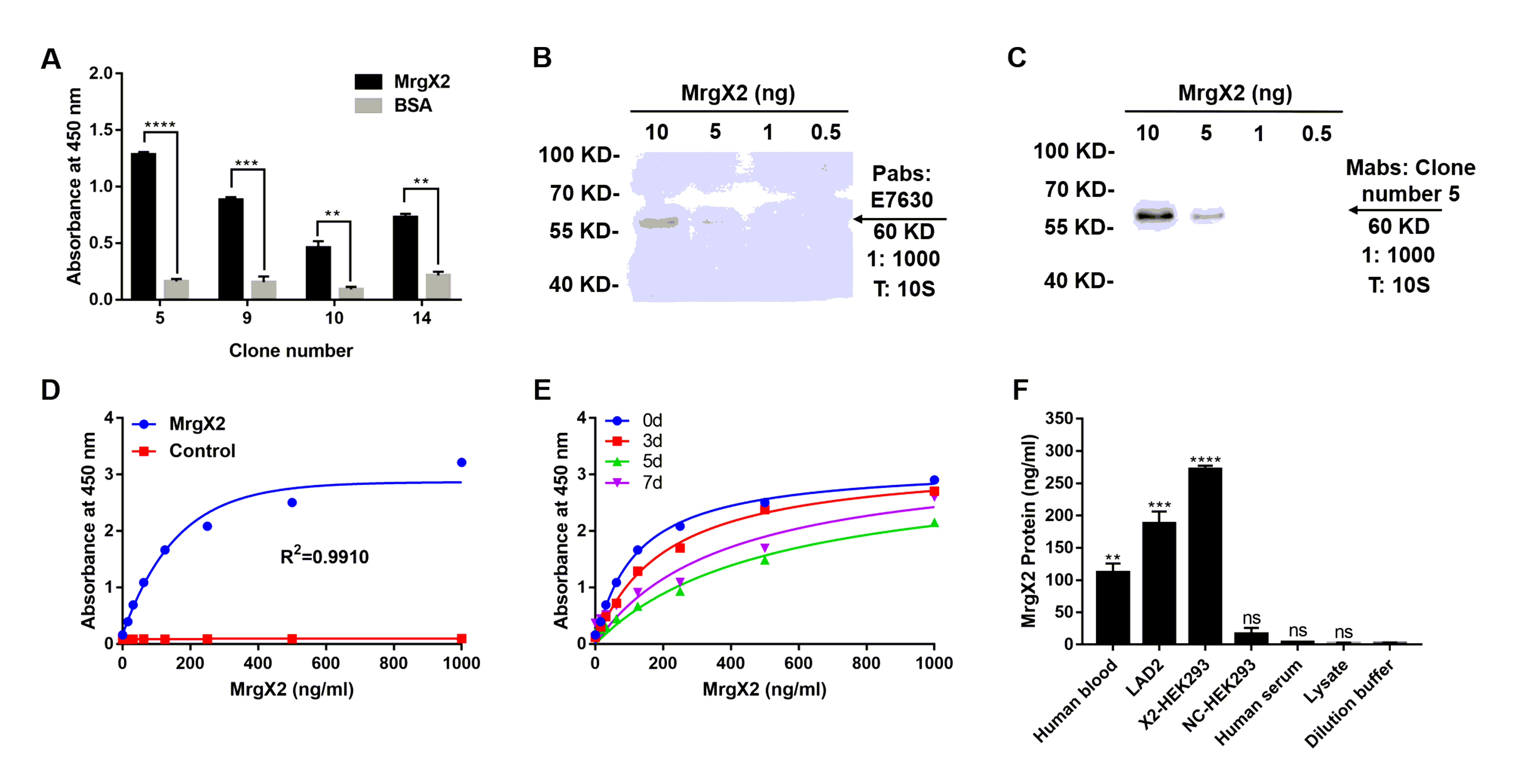

Supplement: Supplementary file 5 — Additional file 5: Fig. S5. Establishment and methodological investigation of human MrgX2-ELISA. a Double antibody sandwich screening of matched antibodies. Student’s t test (nonparametric tests) was used to determine statistical significance. b Western blot to examine the specificity of E7630 rabbit polyclonal purified antibody. c Western blot to investigate the specificity of mouse monoclonal purified antibody. d Human MrgX2-Inspection of standard curve of ELISA. e Investigation of stability of human MrgX2-ELISA. f Investigation of specificity of human MrgX2-ELISA. One-way analysis of variance (Bonferroni’s multiple comparisons test) was used to determine statistical significance. Data are expressed as mean ± SEM from at least three independent experiments. **p < .01, ***p < .001, ****p < .0001 vs. negative control. [file 13601_2020_361_MOESM5_ESM.png]

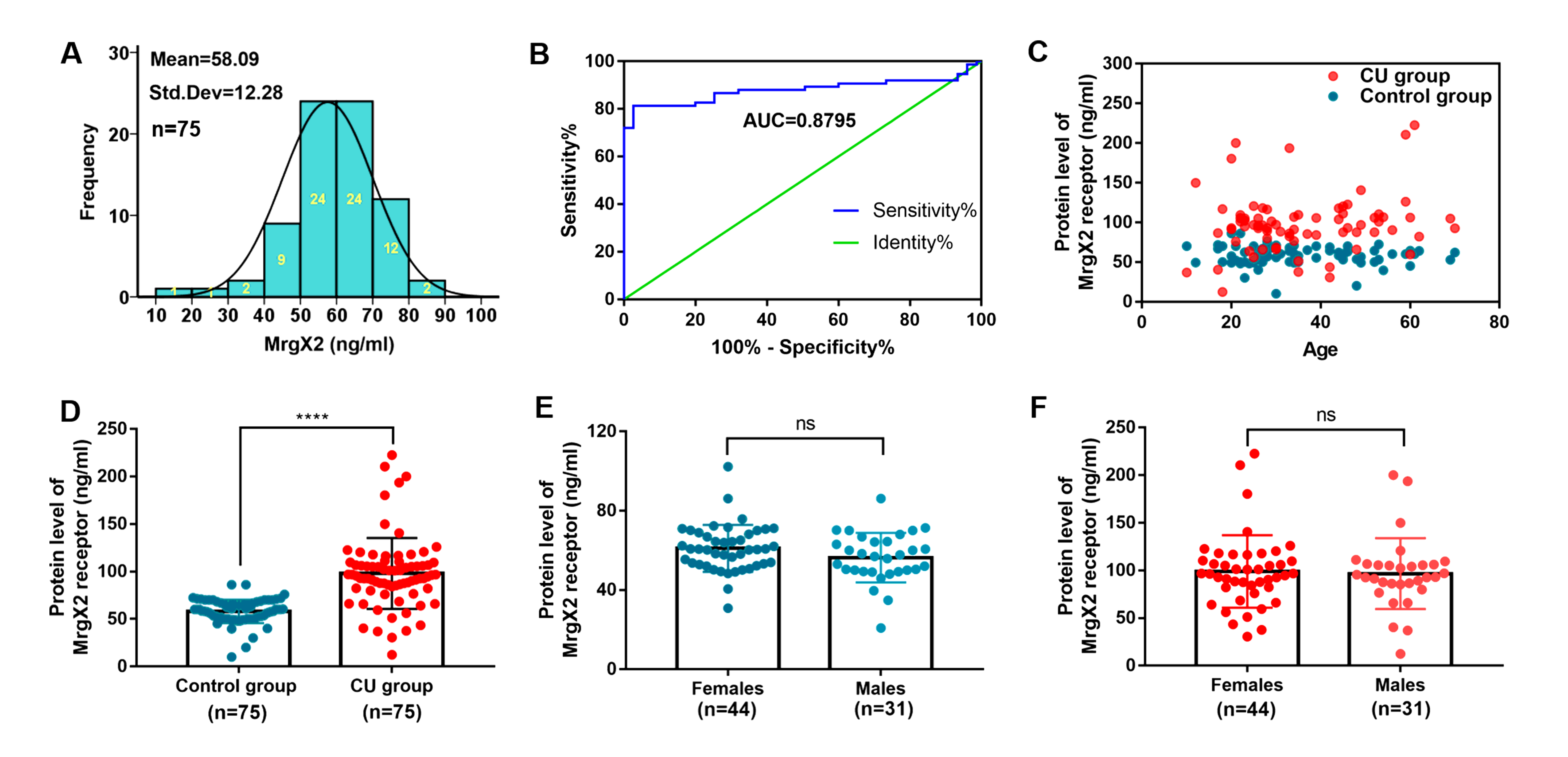

Supplement: Supplementary file 6 — Additional file 6: Fig. S6. Clinical application of human MrgX2-ELISA. a Healthy subjects frequency distribution of human blood MrgX2 concentration in the population (n = 75). b ROC curve of human MrgX2 protein expression (n = 150), green line represents the diagnostic reference line; blue line represents the ROC curve of MrgX2. c Scatter plot of blood MrgX2 concentration in CU patients (n = 75) and healthy subjects (n = 75). d Histogram of blood MrgX2 concentration in CU patients (n = 75) and healthy subjects (n = 75). e Comparison of blood MrgX2 concentration in healthy male (n = 31) and healthy famale (n = 44). f Comparison of blood MrgX2 concentration in CU male (n = 31) and CU female (n = 44). Student’s t test (nonparametric tests) was used to determine statistical significance. Data are expressed as mean ± SEM from at least three independent experiments. ****p < .0001 vs. control group. [file 13601_2020_361_MOESM6_ESM.png]
