# Supplementary material for: A novel ELISA method to determine human MrgX2 in chronic urticaria
Source: Clin Transl Allergy. 2020 Dec 9;10:61. doi: 10.1186/s13601-020-00361-8 (PMC7727259; doi:10.1186/s13601-020-00361-8)
Supplement: Supplementary file 7 — Additional file 7: Table S1 Detection line, quantitative limit, linear range and accuracy. Table S2 Inter-Assay and Intra-Assay Precision. Table S3 MrgX2 concentrations for groups of healthy subjects and CU patients. Table S4 Comparisons of MrgX2 concentrations between respective groups of healthy subjects and CU patients. [file 13601_2020_361_MOESM7_ESM.docx]

**A novel ELISA method to determine human MrgX2 in chronic urticaria**

Yuanyuan Ding^a^, Tao Zhang^a^, Rui Liu^a^, Delu Che^a^, Nan Wang^a^, Langchong He^a^*

*^a^ College of Pharmacy, Xi’an Jiaotong University, Xi'an710061 China.*

*Corresponding author

Langchong He, Xi’an Jiaotong University, Yanta West Road, Xi’an 710061, China

Tel.: +86-29-82656788; Fax: +86-29-82655451

E-mail: helc@mail.xjtu.edu.cn

**Additional files: Tables:**

Table S1 Detection line, quantitative limit, linear range and accuracy

| Validation  criterion | LOD  ng/mL | LOQ  ng/mL | Linearity range  ng/mL | Accuracy  % Recovery |
| --- | --- | --- | --- | --- |
| MrgX2 | 3.125 | 6.25 | 3.125-200 | 92.37-108.0% |

Table S2 Inter-Assay and Intra-Assay Precision

| Validation  criterion | Precision | | | | | | |
| --- | --- | --- | --- | --- | --- | --- | --- |
|  | Inter-day (n = 5) | | |  | Intra-day (n = 5) | | |
|  | 25  ng/mL | 50  ng/mL | 100 ng/mL |  | 25  ng/mL | 50  ng/mL | 100 ng/mL |
| MrgX2 | 11.88% | 10.82% | 8.984% |  | 1.885% | 9.163% | 4.966% |

Table S3 MrgX2 concentrations for groups of healthy subjects and CU patients ^a^

| Group | n | 25th percentile | Median | 75th  percentile |
| --- | --- | --- | --- | --- |
| Healthy subjects | 75 | 50.11 | 60.00 | 66.80 |
| CU patients | 75 | 82.42 | 95.62 | 109.43 |

^a^For each group of healthy subjects or CU patients, the median, 25th percentile, 75th percentile, and interquartile range were determined.

Table S4 Comparisons of MrgX2 concentrations between respective groups of healthy subjects and CU patients^a^

| Group | *p* |
| --- | --- |
| CU patients (n= 75) vs healthy subjects (n = 75) | <0.0001 |
| healthy male (n=31) vs healthy female (n=44) | 0.1039 |
| CU male (n=31) vs CU female (n=44) | 0.7892 |

^a^ Comparisons of MrgX2 concentrations between healthy subjects and CU patients were performed by using a nonparametric t test.
